# Human Chitinases and Chitinase-Like Proteins as Indicators for Inflammation and Cancer

**Published:** 2007-05-03

**Authors:** Julia Kzhyshkowska, Alexei Gratchev, Sergij Goerdt

**Affiliations:** Department of Dermatology and Allergology, University Medical Centre Mannheim, Ruprecht-Karls, University of Heidelberg, Mannheim D-68167, Germany

**Keywords:** Glyco_18 Domain, Macrophage, Tumor, Arthritis, Gaucher Disease, Asthma

## Abstract

Human Glyco_18 domain-containing proteins constitute a family of chitinases and chitinase-like proteins. Chitotriosidase and AMCase are true enzymes which hydrolyse chitin and have a C-terminal chitin-binding domain. YKL-40, YKL-39, SI-CLP and murine YM1/2 proteins possess solely Glyco_18 domain and do not have the hydrolytic activity. The major sources of Glyco_18 containing proteins are macrophages, neutrophils, epithelial cells, chondrocytes, synovial cells, and cancer cells. Both macrophages and neutrophils use the regulated secretory mechanism for the release of Glyco_18 containing proteins. Glyco_18 containing proteins are established biomarkers for human diseases. Chitotriosidase is overproduced by lipid-laden macrophages and is a major marker for the inherited lysosomal storage Gaucher disease. AMCase and murine lectin YM1 are upregulated in Th2-environment, and enzymatic activity of AMCase contributes to asthma pathogenesis. YKL proteins act as soluble mediators for the cell proliferation and migration, and are also involved in rheumatoid arthritis, inflammatory bowel disease, hepatic fibrosis and cirrhosis. Chitotriosidase and YKL-40 reflect the macrophage activation in atherosclerotic plaques. Serum level of YKL-40 is a diagnostic and prognostic marker for numerous types of solid tumors. YKL-39 is a marker for the activation of chondrocytes and the progression of the osteoarthritis in human. Recently identified SI-CLP is upregulated by Th2 cytokine IL-4 as well as by glucocorticoids. This unique feature of SI-CLP makes it an attractive candidate for the examination of individual sensitivity of patients to glucocorticoid treatment and prediction of side effects of glucocorticoid therapy. Human chitinases and chitinase-like proteins are found in tissues and circulation, and can be detected by non-invasive technologies.

## Family Composition: Domain Organisation, Enzymatic Activity and Lectin Properties

The common feature of mammalian chitinases and chitinase-like proteins is the presence of highly conserved Glyco_18 domain. The Glyco_18 domain is characteristic for the evolutionary conservative chitinases, which belong to the glycosyl hydrolase family 18. Family 18 chitinases are distributed in a wide range of organisms, including bacteria, plants, fungi, insects, viruses, protozoan parasites, and more recently were identified in mammals. Family 18 chitinases catalyze the hydrolysis of chitin via substrate-assisted mechanism. An additional activity of these enzymes is *trans*-glycosylation resulting in the formation of chitin oligomers longer than the initial substrate (Sasaki et al. 2002). After cellulose, chitin is the second most abundant polysaccharide in nature. Chitin is a polymer of *N*-acetylglucosamine that is a component of fungal cell walls, exoskeletons of insects and crustaceans, and microfilarial sheaths of parasitic nematodes. Chitinase production plays a role in the life cycle of chitin-containing fungi and parasites; but there is no endogenous chitin in mammals. Evolutionary conserved function of chitinases in lower life forms is a host-defence against chitin-containing organisms (Elias et al. 2005).

**Figure 1:**
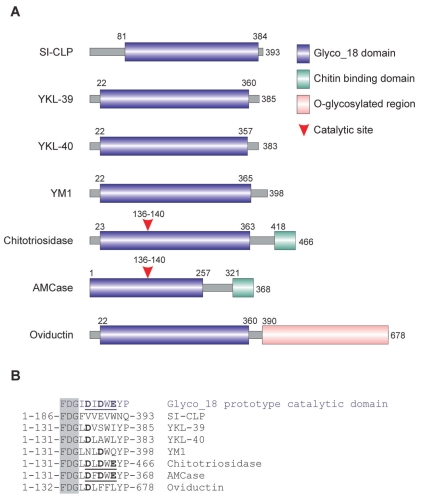
SI-CLP is a novel member of Glyco_18 domain-containing human chitinases and chitinase-like proteins. (**A**) Schematic representation of domain organisation of mammalian Glyco_18 domain-containing protein. (**B**) Critical aminoacid in catalytic sites. The characteristic FDG sequence preceding catalytic motif is shown in shadowed box. Catalytic aminoacids are shown in bold. Complete active catalytic motifs are underlined (Kzhyshkowska et al. 2006). This research was originally published in Blood. Kzhyshkowska, J et al. Novel stabilin-1 interacting chitinase-like protein (SI-CLP) is up-regulated in alternatively activated macrophages and secreted via lysosomal pathway Blood. 2006; 107:3221–3228. © the American Society of Hematology.

There are six Glyco_18 domain-containing proteins identified in human up to date (see Fig. 1A). In addition two closely related Glyco_18 domain-containing proteins, YM1 and YM2, have been reported in rodents. However, there are no human analogues for YM1/YM2 neither on genomic nor on protein level. Only two mammalian Glyco_18 containing proteins possess enzymatic activity: chitotriosidase (Renkema et al. 1995; Boot et al. 1995) and acidic mammalian chitinase AMCase (Boot et al. 2001). Chitotriosidase is able to hydrolyse the artificial substrate 4-MU-chitotrioside as well as chitin (Renkema et al. 1995). In the excess of substrate, chitotriosidase exhibit *trans*-glycosylation activity toward substrate molecules (Aguilera et al. 2003). AMCase is also capable of cleaving both natural chitin from fungal cell wall and crab shell, and artificial chitin-like substrates. In contrast to chitotriosidase, AMCase is extremely acid-stable and shows second pH optimum around pH 2 (Boot et al. 2001). The catalytic activity of Glyco_18 domain-proteins is defined by the presence of a catalytic glutamic acid in the context of a DXDXE site located directly (with one aminoacid as a spacer) after the characteristic aminoacid triplet FDG (Fusetti et al. 2002). Both chitotriosidase and AMCase in addition to Glyco_18 domain, contain the functional chitin-binding domain on their C-terminus.

YKL-39, YKL-40, SI-CLP and YM1/YM2 are composed solely out of Glyco_18 domain. Oviductin/MUC9 contains a Glyco_18 domain and a long fragment with numerous sites for *O*-glycosylation, characteristic of mucins. YKL-39, YKL-40, SI-CLP, YM1/YM2 and oviductin lack critical aminoacids within the catalytic site (Fig. 1B) and do not exhibit enzymatic activity. The exchange of glutamic residues in catalytic centre of chitotriosidase to leucine, which is present in YKL-40 in the same position, converts the catalytically active site into chitin-binding site (Renkema et al. 1998). Accordingly, YKL-40 was established to be a chitin-specific lectin (Renkema et al. 1998). Binding of chitin fragments of different lengths identified nine sugar binding subsites in the groove in YKL-40, and showed that the specificity of its chitin-binding depends on the length of oligosaccharides (Fusetti et al. 2003). Crystal structures of native YKL-40 and its complex with GLcNAc8 showed that YKL-40 binds to oligosaccharide ligand in a fashion identical to catalytically active family 18 chitinases. But in contrast to chitinases, in YKL-40 oligosaccharide ligand induces large conformational change that might have regulatory significance during host response to fungi or nematodes (Houston et al. 2003). Although sugar binding was not examined for all mammalian chitinase-like proteins, it can be predicted that the presence of enzymatically inactive Glyco_18 domain is indicative for lectin properties. Currently the enzymatically inactive chitinase-like proteins are considered to constitute a novel class of cytokines with lectin properties.

## Cell Type Specificity and Regulation of Expression

The major cell types producing mammalian chitinases and chitinase-like proteins are macrophages, neutrophils, epithelial cells, chondrocytes and synovial cells, as well as tumor cells (for summary see Table 1). Expression of these proteins is regulated on the mRNA level by various cytokines and hormones.

Chitotriosidase (CHIT1) and YKL-40 (CHI3L1, HC gp-39) were the first human Glyco_18 containing proteins identified and are the best characterised from the biological and clinical points of view.

The sources of secreted chitotriosidase are abnormal lipid-laden macrophages formed in tissues of Gaucher disease patients. Gaucher cells can be classified as a variation of alternatively activated macrophages; they express CD68, C14, HLA class II, CD163, CCL18, and IL-1-receptor antagonist, but do not express CD11b, CD40, and proinflammatory cytokines such as TNFα and MCP1 (Boven et al. 2004). In vitro, monocyte-derived macrophages start to produce chitotriosidase approximately after 7 days of culturing (Boot et al. 1999). Production of chitotriosidase in cultured macrophages is strongly stimulated by GM-CSF (van Eijk et al. 2005). The same study demonstrates the inhibitory effect of IFNγ and IL-4 on expression of chitotriosidase. At the same time several reports indicate the stimulatory effect of proinflammatory stimuli (IFNγ, TNFα, LPS) on the expression of chitotriosidase in monocyte-derived macrophages (Di Rosa et al. 2005; Malaguarnera et al. 2005). The discrepances might be explained by differences in the generation of macrophage cultures and can reflect the contribution of secondary processes, which occur in macrophage cultures. Careful studies on the chitotriosidase promoter are required to dissect the mechanisms regulating its expression. Two enzymatically active isoforms of chitotriosidase, 50 kDa and 39 kDa were originally purified out of spleen of a Gaucher patient (Renkema et al. 1995). The 39 kDA isoform lacks C-terminal chitin-binding domain and represent a truncated form of the full-size protein (Renkema et al. 1997). It is formed during the post-translational processing. In addition the minor 40-kDa isoform is formed as a result of alternative splicing. The 50 kDa is the predominantly secreted isoform, but all the three isoforms are formed in macrophages in vitro and can be detected by proteomics techniques in serum of Gaucher patients (Renkema et al. 1997; Quintana et al. 2006). In healthy human organism mRNA for chitotriosidase was found in lymph nodes, lung, and bone marrow, and its expression is restricted to the professional phagocytes (Boot et al. 2005b). In addition to macrophages, neutrophils were shown to be a source of chitotriosidase in human (van Eijk et al. 2005). In contrast, mRNA for murine chitotriosidase is expressed in the gastrointestinal tract, the tongue, fore-stomach and Paneth cells in the small intestine (Boot et al. 2005b).

Both chitotriosidase and YKL-40 were found to be markers for activated macrophages in atherosclerotic plaque (Boot et al. 1999). Interestingly, chitotriosidase and YKL-40 are expressed in distinct macrophage subpopulation in atherosclerotic lesions indicating the involvement of differentially polarised macrophages in the progression of atherosclerosis. In vitro, the same macrophages, which secrete chitotriosidase, can secrete YKL-40 (Renkema et al. 1998). YKL-40 mRNA was detected after 2 days of in vitro cultivation of macrophages and reached the maximum at day 14, while expression of chitotriosidase started after 7 days and dramatically increase with time. The authors concluded that the expression of YKL-40 and chitotriosidase is regulated independently. YKL-40 is also expressed in human neutrophils, which release YKL-40 in inflammatory conditions (Volck et al. 1998).

YKL-40 was originally isolated as one of the major secreted proteins of cultured human articular chondrocytes and synovial fibroblasts (Hakala et al. 1993). The name YKL-40 derives from the on letter code for the tyrosine, lysine and leucine in its N-terminus and its apparent molecular weight (Hakala et al. 1993). Later YKL-40 was shown to be a differentiation marker for macrophages, where its expression is predominantly regulated by the transcription factor SP1 (Rehli et al. 1997, 2003). YKL-40 is secreted in vitro by numerous human cancer cell lines of different origin, including glioblastoma, colon cancer, ovarian cancer, prostate cancer, osteosarcoma, malignant melanoma (Johansen et al. 2006). In vivo, YKL-40 expression was found in places with intensive tissue remodelling. It is not generally synthesised by healthy human chondrocytes in vivo, however, mRNA for YKL-40 was detected in osteoarthritic cartilage (Hakala et al. 1993; Connor et al. 2000). Immunohistochemical analysis showed that YKL-40 protein is expressed in chondrocytes of osteoarthritic cartilage mainly in the superficial and middle zone of the cartilage rather than the deep zone (Volck et al. 1999). YKL-40 protein expression was detected in biopsies of glioblastomas, breast and colon cancer (Nutt et al. 2005; Pelloski et al. 2005; Johansen et al. 2006).

**Figure 2:**
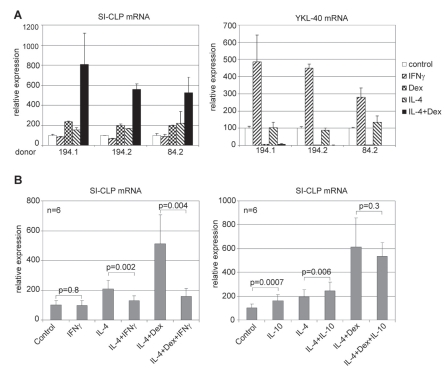
Real-time RT-PCR analysis of SI-CLP and YKL-40 expression in human macrophages. Peripheral blood derived monocytes non-stimulated (control) or stimulated with cytokines as indicated were propagated in culture for 6 days. Three representative donors with differential responsivities are presented. (**A**) IL-4, dexamethasone and combination of both induce SI-CLP mRNA upregulation in macrophage cultures, the lowest SI-CLP expression is detected in case of IFNγ stimulation. (**B**) IFNγ induces YKL-40 mRNA expression, whereas dexamethasone has strong inhibitory effect (Kzhyshkowska et al. 2006). This research was originally published in Blood. Kzhyshkowska, J et al. Novel stabilin-1 interacting chitinase-like protein (SI-CLP) is up-regulated in alternatively activated macrophages and secreted via lysosomal pathway Blood. 2006; 107:3221–3228. © the American Society of Hematology.

Various hormones and cytokines regulate the production of YKL-40. Studies in IL-6 knockout mice reveled that YKL-40 expression depends on IL-6 (Johansen et al. 2006). Expression of YKL-40 mRNA in human monocyte is strongly stimulated by IFNγ, and inhibited by IL-4 and dexamethasone (Fig. 2(Kzhyshkowska et al. 2006b)). Proinflammatory hormones arg-vasopressin (AVP) and parathyroid hormone-related protein (PTHrP) differentially affect the secretion of YKL-40 by cultured chondrocytes (Petersson et al. 2006). Both PTHrP and AVP stimulated the secretion of YKL-40 from chondrocytes derived from patients with rheumatoid arthritis. In contrast AVP decreased and PTHrP did not affect the secretion of YKL-40 from chondrocytes from patients with osteoarthritis. The chondrocytes derived from the healthy subjects showed that AVP had an inhibitory effect, whereas the PTHrP did not change the amounts of YKL-40.

In contrast to the broad range of YKL-40 expression, YKL-39 production is predominantly demonstrated for chondrocytes and synoviocytes (Hu et al. 1996). Comparative quantitative analysis using real-time RT-PCR demonstrated that YKL-40 was strongly upregulated by IFNγ, while YKL-39 was expressed on a very low level in all macrophage subpopulation tested and no specific effects of IFNγ, IL-4 or dexamethasone were detected (Fig. 2) (Kzhyshkowska et al. 2006b). To our knowledge there is only one report showing that YKL-39 can be expressed by macrophages. Messenger RNAs for both YKL-39 and YKL-40 were strongly upregulated in the brain of patients with Alzheimer’s disease, and this fact was attributed to the alternative activation of microglial macrophage during the course of the disease (Colton et al. 2006).

Using Northern blot analysis it was demonstrated that the second enzymatically active mammalian chitinase, AMCase is expressed in gastrointestinal tract and to the less extent in the lung (Boot et al. 2001). Significant increase of AMCase mRNA and protein was detected in lungs of ovalbumin-sensitised mice (aeroallergen asthma model) (Zhu et al. 2004). Immunohistochemical examination showed that the AMCase is expressed in both epithelial cells and macrophages in lungs of sensitised mice. Expression of AMCase was preferentially stimulated by Th2 cytokine IL-13, and to much less extent by IL-4, while IL-11 and IL-10 did not result in increase of AMCase expression (Zhu et al. 2004). IL-13 did not induce the expression of chitotriosidase in the same model. Theability of AMCase to diminish Th2-inflammation and dependance of this effect on IL-13 receptor and STAT6 suggested that the AMCase acts downstream of IL-13. In situ hybridisation with human AMCase probes showed that the AMCase mRNA is induced in both epithelial cells and subepithelial cells (probably macrophages) in lung tissues of asthmatic patients (Zhu et al. 2004).

YM1 and YM2 were found only in rodents and have no human analogue neither on genomic nor on protein level (Chang et al. 2001; Raes et al. 2005). YM1 is predominantly produced by macrophages (Chang et al. 2001; Welch et al. 2002; Nair et al. 2003, 2005). In murine models of Th2-type parasite infections, expression of both YM-1 and AMCase depends on the activity of IL-21 receptor, which has structural homology to IL-4Rα chain and responds to the Th2 cytokine IL-21 (Pesce et al. 2006).

Since both YM1 and AMCase are induced in Th2-environment, several researchers consider human AMCase to be a functional homologue of murine YM1. However, both absence of chitin-binding domain and catalytic aminoacids within Glyco_18 domain of YM1 (see Fig. 1) argue against this point of view (Boot et al. 2005a). Moreover in murine experimental model of asthma, YM1 and AMCase were shown to be useful markers for distinguishing between proximal and distal airway epithelium. AMCase is expressed by non-mucus-producing CCSP-expressing cells of the distal airway, and YM-1 is expressed by the mucus-producing cells of the proximal airways (Homer et al. 2006).

We have recently demonstrated that the human macrophages produce one more catalytically inactive Glyco_18 domain-containing protein, SI-CLP (stabilin-1 interacting chitinase-like protein) (Kzhyshkowska et al. 2006b). The expression of SI-CLP is induced by Th2 cytokine IL-4 and glucocorticoid dexamethasone (Fig. 2). Combined IL-4/dexamethasone stimulation has a synergistic effect and is impaired in the presence of prototype Th1 cytokine IFNγ. In vivo, high amounts of SI-CLP were detected in macrophages from bronchoalveolar lavage of patients with chronic airway inflammation. SI-CLP protein expression is not restricted to primary macrophages and is found in Raji cells, Jurkat cells, various tumor cell lines (Kzhyshkowska et al. 2006b), as well as in CD3 + T-cells, isolated from peripheral blood of healthy donors (Kzhyshkowska, unpublished data). However, until now the secretion of SI-CLP was shown only for IL-4-stimulated macrophages (Kzhyshkowska et al. 2006b).

The most distant member of human Glyco_18 domain-containing proteins, oviductin (mucin 9, MUC9; oviduct-specific glycoprotein, OGP) is exclusively expressed and secreted by oviductal epithelium (Malette et al. 1995; Arias et al. 1994; Lagow et al. 1999). Estradiol and luteinizing hormone both have a stimulatory effect on oviductin mRNA in fertile women. However, individual contribution of these stimulations was difficult to determine since the luteinizing hormone surge is dependent on the estradiol increase. In the same study progesterone was shown to have a clear inhibitory effect on oviductin mRNA expression (Briton-Jones et al. 2001). Direct stimulatory effect of estradiol-17 β was demonstrated for the murine oviductin promoter (Takahashi et al. 2000).

## Intracellular Sorting and Modes of Secretion

Up to date two types of vesicles were demonstrated to secrete chitinases and chitinase-like proteins: lysosomes in macrophages and secretory granula in neutrophils.

Intracellular localisation studies demonstrated that the chitotriosidase and SI-CLP are primarily localised in lysosomes in macrophages (Renkema et al. 1997; Kzhyshkowska et al. 2006b). Besides the classical, constitutively operating ER/Golgi secretory pathway, which is primarily regulated on the level of gene expression, macrophages use non-classical (Nickel, 2003) and lysosomal secretory pathways (Andrews, 2000; Stinchcombe and Griffiths, 1999). The lysosomal secretion route in macrophages is regulated by specific sorting of newly synthesised products into secretory lysosomes (Logan et al. 2003). Constitutive delivery of lysosomal enzymes to the lysosomes is ubiquitously mediated by mannose-phosphate receptors CI-MPR and CD-MPR (Ghosh et al. 2003; Kornfeld and Mellman, 1989; Kornfeld, 1986). However, Glyco_18 domain-containing proteins differ from other lysosomal enzymes by the lack of *N*-glycosylation typical of lysosomal enzymes and necessary for the recognition by MPRs (Renkema et al. 1997; Boot et al. 2001). Thus, lysosomal routing of Glyco_18 containing proteins can differ from the classical MPR-mediated one. We have recently demonstrated that the intracellular sorting of SI-CLP in alternatively activated macrophages is mediated by the scavenger receptor stabilin-1. (Kzhyshkowska et al. 2006b) SI-CLP is recognised by stabilin-1 in *trans*-Golgi network and delivered to the late endosomes and consequently into Lamp1-positive and secretion-committed CD63-positive lysosomes. It is currently unknown whether the stabilin-1 can mediate lysosomal routing of other than SI-CLP chitinase-like proteins, and which other intracellular receptors can be involved in this process.

Synthesis, processing and intracellular sorting of human chitotriosidase was examined in details in primary monocyte derived macrophages, which were differentiated in culture in the absence of Th1 or Th2 cytokines (Renkema et al. 1997). Immunoelectron microscopy and subcellular fractionation demonstrated that chitotriosidase is sorted into lysosomes. Chitotriosidase was detected in lysosomal vesicles comparably to cathepsin D, and chitotriosidase activity was found in fractions positive for lysosomal enzyme β-hexoaminidase. Chitotriosidase was shown to be synthesised as a 50 kDa protein and partially processed resulting in a 39 kDa form. The 39-kDa isoform was predominantly found in dense core fractions of mature lysosomes, while the 50-kDa form was found in fractions of lower density, which were considered by the authors to contain pre-lysosomal compartment (Renkema et al. 1997). However, it can be also hypothesised that the 50-kDa form was sorted into a distinct subpopulation of secretory-committed lysosomes. In the bloodstream, the 50 kDa isoform was predominant, while the 39-kDa isoform was abundant in the tissues (Renkema et al. 1997).

Peripheral blood monocytes differentiated in culture into mature macrophages secrete both chitotriosidase and YKL-40 (Renkema et al. 1998). However, no intracellular localisation studies were performed up to date to define whether YKL-40 and chitotriosidase are sorted into the same vesicles in macrophages.

In neutrophils, both the chitotriosidase and YKL-40 are sorted into specific granules (Volck et al. 1998; Nordenbaek et al. 1999; van Eijk et al. 2005). Differential induction of lysosomal or secretory granula secretion in polymorphonuclear neutrophils (PMN) demonstrated that chitotriosidase is not sorted into lysosome-like azurophilic granules. Using immunogold double labelling experiments, chitotriosidase was detected in specific lactoferrin-containing granules in PMN (van Eijk et al. 2005). YKL-40 also co-localises with lactoferrin, but not with gelatinase in both stimulated and non-stimulated neutrophils. It is suggested that neutrophil-released YKL-40 acts as an autoantigen in rheumatoid arthritis (RA). Moreover, release of YKL-40 from specific neutrophil granules was suggested to lead to the post-transfusional complications and can be prevented by the pre-storage leukocyte depletion by filtration of whole blood (Cintin et al. 2001).

The current picture of the intracellular localisation and regulation of chitinase and chitinase-like proteins is far from being complete. Particularly, it is not known, whether these proteins are sorted into a specific subclass of lysosomes in macrophages, and whether there is a mechanism for the selective secretion of these lysosomes. As well, the mechanism of secretion of chitinase and chitinase-like proteins from cells of non-myeloid origin, like fibroblasts and epithelial cells was not studied.

## Glyco_18 Containing Proteins in Human Disorders

Mammalian chitinases and chitinase-like proteins are secreted into extracellular space in tissues and blood circulation. They possess numerous of cell physiological and immunomodulatory activities (Table 2). Accumulating data point toward connection of their biological activities with various human diseases. Already established association of mammalian Glyco_18 domain-containing proteins with various human disorders is summarised in Table 3.

### Chitotriosidase

Chitotriosidase (ChT), is a human analogue of chitinases from non-vertebrate species that serves both as a diagnostic hallmark of Gaucher disease (GD, glucosylceramidosis), and as a marker for monitoring the efficacy of various therapeutic approaches for treatment of GD (Hollak et al. 1994, 2001; de Fost et al. 2006; Pastores and Barnett, 2005; Pastores et al. 2005). Gaucher disease is caused by recessively inherited deficiency in activity of lysosomal hydrolase—glucocerebrosidase, and is characterised by accumulation of glucosylceramide (glucocerebroside) in the lysosomes of macrophages (Bussink et al. 2006). This process is restricted to tissue macrophages that transform into large swollen lipid-laden Gaucher cells. Pathological consequences of the accumulation of lipid-laden macrophages include hepatosplenomegaly, bone lesions, and less frequently neurological abnormalities. Limited correlation between genotype and phenotype of the GD, as well as highly expensive enzyme supplementation therapy indicated the necessity of easily identifiable biomarker for the GD. In 1994, Hollak et al. reported, that the median activity of chitotriosidase in plasma samples of GD patients is more than 600 times higher in comparison to control subjects (Hollak et al. 1994). Activity of chitotriosidase in plasma is a surrogate marker for the tissue Gaucher cells. Accurate monitoring of chitotriosidase is performed by determination of hydrolytic activity towards fluorogenic chitooligosacharide substrates that has been improved recently (Aguilera et al. 2003). Proteomics approach demonstrated the possibility for the quantitative imaging of various chitotriosidase isoforms (Quintana et al. 2006). Plasma chitotriosidase activity strongly correlated with integrated intensities of chitotriosidase isoforms. But no methods are available at the moment for the assessment of individual contribution of each chitotriosidase isoform in total activity. Comparison of chitotriosidase activity in urine and plasma of GD patients demonstrated that the enzyme activity in urine does not correlate well with the plasma values. The authors suggested that the urinary chitotriosidase reflects the renal pathology rather than Gaucher cell activity. In sharp contrast to chitotriosidase, the levels of chemokine CCL18 both in plasma and in urine correlated perfectly with other GD values. Taking under consideration the existence of recessively inherited deficiency in chitotriosidase with an incidence of about 6% in Caucasian (Boot et al. 1998), CCL18 can be used as an alternative biomarker for enzyme-deficient GD patients (Boot et al. 2004,^2006^; Deegan et al. 2005).

Degree of chitotriosidase activity in plasma can be also applied for the evaluation of likelihood of lysosomal storage disorders other than GD (Guo et al. 1995; Ries et al. 2006; Wajner et al. 2004; Aerts et al. 2005). The examination of chitotriosidase activity provides useful information for the selection of further confirmatory assays for sphingomyelinase deficiency (SMD, Niemann-Pick disease type A/B) and Niemann-Pick disease type C (NPC) (Ries et al. 2006). Chitotriosidase is the only biomarker identified up to date for the monitoring of lipid-laden macrophages and the efficacy of enzyme-replacement therapy in male Fabry patients. Fabry disease is a X-linked globotriaosylceramidosis caused by the deficiency in the lysosomal α-galactosidase A (AGA) (Vedder et al. 2006). Plasma chitotriosidase can be detected in very young age, precedes clinical manifestations and is elevated constituently with the accumulation of lipid-laden macrophages in tissues. Therapy with recombinant AGA results in normalisation of plasma chitotriosidase levels (Vedder et al. 2006). Comparable with GD patients elevation of chitotriosidase activity was demonstrated in plasma of patients with β-thalassemia, a haematological disorder caused by genetic defect of the synthesis of β-globin chains leading to unproductive erythropoiesis and enormous expansion of reticuloendothelial system (Barone et al. 1999; Altarescu et al. 2002). It was proposed that iron-mediated damage of lysosomal system and macrophage activation may result in increased secretion of chitotriosidase (Barone et al. 1999).

Expression of chitotriosidase mRNA is induced in patients with non-alcoholic fatty liver disease —steatohepatitis (NASH) (Malaguarnera et al. 2006a). NASH comprises a wide spectrum of liver damages ranging from uncomplicated steatosis to advanced fibrosis and cirrhosis. NASH can occur, for example, in association with use of drugs, surgical interventions, and metabolic disorders (Malaguarnera et al. 2006b). Using fractionation of liver cells the authors demonstrated that overproduction of chitotriosidase occurs solely in Kupffer cells (liver macrophages). Overproduction of chitotriosidase resulted in the activation of hepatic stellate cell, suggesting the role of this conservative enzyme in the progression of hepatic fibrosis (Malaguarnera et al. 2006a, 2006b).

Elevated expression of chitotriosidase mRNA was detected in Alzheimer’s disease (AD), and was even more pronounced in Ischemic cerebrovascular dementia (CvD) (Di Rosa et al. 2006). AD is a progressive neurodegenerative disorder, characterised by the formation of senile plaque composed of insoluble β-amiloid fibril deposits, astrocytes and degenerating neurons. Pathophysiology of CvD includes the development of brain ischemia and multi-infarct cognitive impairment. Despite the heterogenous nature, there are common inflammatory processes, i.e. activation microglia and T-cell immunity, occurring during the progress of AD and CvD. Induction of chitotriosidase gene expression in parallel with other cytokines is a marker for the inflammatory processes during the neurodegenerative disorders. Moreover, chitotriosidase was reported to be a specific marker for macrophage activation occurring in stroke. Chitotriosidase activity correlates with stroke severity independently on preexisting inflammatory or infectious conditions (Sotgiu et al. 2005). The role of this enzyme in the pathology of brain has to be further investigated.

Elevated levels of serum chitotriosidase were also found in disorders caused by the abnormal activation of immune system, including sarcoidosis (Grosso et al. 2004) and atherosclerosis (Boot et al. 1999; Artieda et al. 2003). In atherosclerosis, chitotriosidase is produced by subsets of macrophages in atherosclerotic plaque, and enzyme activity is elevated up to 55-fold in extracts of atherosclerotic tissue. It is hypothesised that the chitotriosidase together with YKL-40 regulate cell migration and tissue remodelling during atherogenesis (Boot et al. 1999).

Human chitotriosidase also associates with pathogen-driven diseases, and in particular with fungal infections, suggesting the role of this enzyme in host-defence against chitin-containing pathogens (Labadaridis et al. 1998, 2005). Thus, the elevated level of chitotriosidase activity was detected in plasma and/or urine of neonates with fungal infection (Labadaridis et al. 2005). Increased activity of chitotriosidase was also found in neonates with bacterial infections, suggesting that it is rather an indicator of macrophage activity during the course of infectious diseases, but not a specific anti-fungal response. The authors proposed that the identification of an increased level of chitotriosidase activity can be used as an indicator for possible fungal infection if the bacterial infection is absent. Furthermore, serial urine analysis can be useful for the monitoring of the efficiency of anti-fungal therapy in neonates (Labadaridis et al. 2005). Demonstrated by van Eijk et al. anti-fungal activity of human chitotriosidase both in vitro and in the animal model, indicates its role as an innate immunity component in host response against chitin-containing pathogens (van Eijk et al. 2005).

Plasma chitotriosidase activity is increased in African children with acute *Plasmodium falciparum* malaria and correlates with other disease parameters (Barone et al. 2003). The levels of chitotriosidase were found to be higher in African patients than in Caucasian population. Interestingly, significant correlation was found between plasma chitotriosidase and reticulo-endothelial activation, as judged by thrombocytopenia degree and serum ferritin level in children with acute malaria.

Thus, the chitotriosidase is indicator for macrophage-driven inflammatory processes in various organs. Its role as an innate immune component in anti-fungal response has to be further confirmed. Chitotriosidase remains to be the main biomarker for the diagnostic and evaluation of therapeutic approaches in GD; however, the additional improvement can be achieved by the parallel use of other markers, like, for example, CCL18.

### YKL-40

YKL-40, also called human cartilage glycoprotein-39 (HC gp-39), is the best investigated human chitinase-like protein regarding its biological activity and association with various disorders. Two comprehensive reviews have been published recently which elucidate in details association of YKL-40 with human disorders, methods of YKL-40 detection and evaluation of YKL-40 as a diagnostic and prognostic factor (Johansen et al. 2006; Rathcke and Vestergaard, 2006). Here, we discuss only the most important biological functions of YKL-40 and briefly summarise major YKL-40- associated pathologies.

Biological activities of YKL-40 include regulation of cell proliferation, adhesion, migration and activation. YKL-40 promotes the growth of human synovial cells, skin and foetal lung fibroblasts. The proliferative effect of YKL-40 synergizes with the effect of insulin-like growth factor-1 (Recklies et al. 2002). At the same time YKL-40 is able to suppress the TNFα and IL-1-induced secretion of matrix metalloproteases and IL-8 in both human skin fibroblasts and articular chondrocytes. Thus, YKL-40 promotes the proliferation and antagonizes catabolic or degradative processes during the inflammatory response of connective tissues (Ling and Recklies, 2004). Cg-Clp1, the conserved molluscan homologue of YKL-40, stimulates proliferation and regulates synthesis of extracellular matrix components in rabbit articular chondrocytes (Badariotti et al. 2006). Recently, the existence of three isoforms YKL-40 was reported: a major and minor forms from resorbing cartilage and a third specie from chondrocytes (Bigg et al. 2006). Affinity chromatography experiments with purified YKL-40 demonstrated specific binding of all three isoforms to collagens types I, II, and III. The chondrocyte-derived YKL-40 isoform was able to prevent collagenolytic cleavage of type I collagen and to stimulate the rate of type I collagen fibril formation. By contrast, the cartilage major form had an inhibitory effect on type I collagen fibrillogenesis. Differential modulatory effect of various YKL-40 isoforms indicates that expression of specific isoforms has to be taken into consideration in the epidemiological and functional studies.

YKL-40 has also cell-type specific effect on cell migration. It induces the migration of vascular smooth muscle cells (VSMC), but not fibroblasts. Moreover, YKL-40 promotes the attachment and spreading of VSMC (Nishikawa and Millis, 2003). The ability of YKL-40 to regulate cell proliferation, adhesion, migration, and activation, as well as to regulate extracellular matrix assembly, correlates well with elevated level of YKL-40 in the sites of chronic inflammation and active connective tissue turnover.

Circulating YKL-40 can be detected by non-invasive methods in human serum or plasma using in-house RIA (Johansen et al. 1993, 2006) as well as by commercially available ELISA (Quidel, Santa Clara, CA (Harvey et al. 1998). Increased concentrations of YKL-40 were detected not only in sites of inflammation, but also in serum of patients with rheumatoid arthritis (RA). In RA, YKL-40 acts as an autoantigen. In contrast to healthy individuals, who show strong bias to regulatory response to YKL-40, 50% of patients with RA exhibit polarisation towards Th1 phenotype (van Bilsen et al. 2004). Local release of YKL-40 in the arthritic joint is followed by a secondary increase of YKL-40 concentration in serum. Several independent groups demonstrated that the elevated levels of YKL-40 in serum reflect the degree of the synovial inflammation and joint destruction in patients with RA and OA (Johansen et al. 1999, 2001; Matsumoto and Tsurumoto, 2001; Peltomaa et al. 2001; Conrozier et al. 2000). Rheumatic symptoms are also common for extra-intestinal manifestations of inflammatory bowel disease (IBL), and elevated level of YKL-40 is a marker for joint involvement in IBL (Bernardi et al. 2003) and for the activity of the disease (Vind et al. 2003).

YKL-40 is expressed in human smooth muscle cells and upregulated in distinct subsets of macrophages in the atherosclerotic plaque. Expression of YKL-40 mRNA was higher in macrophages in early atherosclerotic lesions and in macrophages which infiltrated deep in the lesion and (Boot et al. 1999). YKL-40 is a vascular cell adhesion and migration factor that may have a role in processes leading to vascular occlusion and heart development (Nishikawa and Millis, 2003). In concentration of 1 ng/ml YKL-40 has a profound effect on migration of vascular smooth muscle cell (VSMC), but not on migration of fibroblasts. In addition YKL-40 promotes VSMC attachment and spreading. Proteomics study identified elevated levels of YKL-40 in supernatants of macrophage cell line THP-1 treated with oxidised LDL (in vitro “foam cell“ model) (Fach et al. 2004). Consequently, YKL-40 expression is indicative for the differentiation of macrophages during formation of atherosclerotic plaque (Rathcke and Vestergaard, 2006). Most recently, elevated levels of YKL-40 were found in patients with type 2 diabetes, where YKL-40 positively correlates with insulin resistance and with features of dyslipidaemia. Both YKL-40 and hsCRP were found to be related to insulin resistance in patients with T2D; however, the increase of plasma concentrations of YKL-40 and hsCRP occurred independently (Rathcke et al. 2006). In contrast, in rheumatoid arthritis the serum levels of YKL-40 positively correlated with serum levels of IL-6 and CRP. YKL-40 was suggested to be an independent biomarker for the inflammatory/atherosclerotic processes in T2D patients (Rathcke and Vestergaard, 2006; Rathcke et al. 2006).

YKL-40 was also found to be associated with another disease characterised by chronic inflammation, i.e. pulmonary sarcoidosis. Both macrophages and giant cells in pulmonary sarcoid granuloma express YKL-40, and serum levels of YKL-40 are indicative for sarcoid disease activity and ongoing fibrosis (Johansen et al. 2005b).

Serum levels of YKL-40 are elevated in patients with pathogen-induced inflammation, including purulent meningitis, pneumonia and endotoxaemia caused by endotoxin of. *Escherichia coli.* (Ostergaard et al. 2002; Nordenbaek et al. 1999; Kronborg et al. 2002; Johansen *et al.* 2005a; Rathcke and Vestergaard, 2006). In both meningitis and pneumonia, YKL-40 is secreted by locally activated macrophages (Ostergaard et al. 2002) and neutrophils (Nordenbaek et al. 1999), and was proposed as a specific supplementary serological marker for the activation of granulocytes and macrophages in inflamed tissues (Rathcke and Vestergaard, 2006).

Increased production of YKL-40 is indication for liver pathology. YKL-40 is differentially upregulated in cirrhotic liver on the end-stage of hepatitis C virus (HCV) induced liver cirrhosis (Shackel et al. 2003). Serum level of YKL-40 correlates with YKL-40 mRNA expression in liver (Kamal et al. 2006), and was suggested to be a useful non-invasive marker for evaluation of the degree of fibrosis as well as efficiency of therapy in patients with HCV-associated liver disorders (Saitou et al. 2005). Increased plasma levels of YKL-40 were also suggested to reflect the progression of liver fibrosis in alcoholics (Tran et al. 2000).

Elevated level of YKL-40 in the circulation was found in number of solid tumors including breast cancer, colorectal cancer, ovarian cancer, glioblastoma, metastatic renal and prostate cancer and malignant melanoma (Johansen et al. 2006). Serum levels of YKL-40 are indicative for the poor prognosis and efficiency of metastatic process. For example, serum level of YKL-40 is an independent marker for the aggressiveness of metastatic breast cancer (Jensen et al. 2003). Increased plasma concentration of YKL-40 is related to poor prognosis and shorter survival in patients with ovarian cancer (Hogdall et al. 2003), colorectal carcinoma (Cintin et al. 2002), metastatic prostate carcinoma (Brasso et al. 2006), and melanoma (Schmidt et al. 2006a, 2006b). YKL-40 was recently proposed as a novel marker for the detection of endometrial cancer (Diefenbach et al. 2006). However, in most solid tumors, the serum concentrations of YKL-40 do not show high sensitivity for identification of primary cancer, and determination of YKL-40 cannot be used as a single screening marker for diagnosis of cancer (Johansen et al. 2006). In addition there is no direct correlation between the expression level of YKL-40 and various tumor antigens (Brasso et al. 2006). Out of six necessary criteria of “tumor marker utility system” (Hayes et al. 1996; Hayes 1998), YKL-40 is positive only for three, and therefore is currently considered to be investigational (Johansen et al. 2006). It is suggested that differential expression of YKL-40 reflects difference in biology of cancer cells which produce YKL-40 or do not produce YKL-40 (Johansen et al. 2006).

However, it is still questionable which cell populations or their combinations are critical for the secretion of YKL-40 in human malignancies. In particular, it is not known, whether tumor cells or tumor-associated macrophages are responsible for the elevation of serum levels of YKL-40 during cancer progression. Thus, the elevated levels of YKL-40 can reflect integrated effect of cancer cell activity and activation status of tumor-associated macrophages.

### YKL-39

YKL-39 is currently recognised as a biochemical marker for the activation of chondrocytes and the progress of the osteoarthritis in human that is more accurate than YKL-40 (Knorr et al. 2003).

YKL-39 was identified as an abundantly secreted protein in primary culture of human articular chondrocytes (Hu et al. 1996). N-Terminal sequencing of YKL-40 containing fraction of conditioned medium revealed the presence of a second closely related protein. In case of YKL-40 the sequence was YKLVCY*Y*T*S*WSQ*Y*R while in case of YKL-39 the sequence was YKLVCY*F*T-*N*WSQ*D*R. Since the new protein also had a YKL motif, it was called YKL-39 in accordance with its apparent molecular weight (*M*_W_). In contrast to YKL-40, YKL-39 is not a glycoprotein. Although the predicted number of aminoacids was slightly larger in YKL-39, the presence of carbohydrate in YKL-40, but not YKL-39 explained the differences in *M*_W_ (Hu et al. 1996). YKL-39 accounted for 4% and YKL-40 for 33% of the secreted protein in chondrocyte-conditioned medium. Despite of the high homology on the protein level (more than 50%), the radioimmunoassay developed for the detection of YKL-40 in serum in tissues, was shown to be very specific and does not detect YKL-39 (Hu et al. 1996).

Comparison of the expression of YKL-39 and YKL-40 in osteoarthritic cartilage revealed that YKL-39 mRNA is significantly upregulated in cartilage of patients with osteoarthritis versus normal subjects. Increased expression of YKL-39 mRNA correlated with the upregulation of collagen 2; while YKL-40 mRNA showed no significant upregulation in OA cartilage (Steck et al. 2002). Another study showed that the normal human chondrocytes express mRNA for both YKL-39 and YKL-40. However, while the expression of YKL-39 was upregulated both in early degenerative and late stage osteoarthritis, the expression of YKL-40 was downregulated during the progression of osteoarthritis (Knorr et al. 2003). Proteomic analysis identified YKL-39, but not YKL-40 to be secreted by human osteoarthritic cartilage in culture (De Ceuninck et al. 2005). Thus, the elevation of YKL-40 in serum and synovial fluid of patients with arthritis might result from its overproduction by synovial cell and macrophages, but not by chondrocytes.

Two biological activities of YKL-39 might contribute to the disease progression. One is the induction of autoimmune response (Sekine et al. 2001; Tsuruha et al. 2002; Du et al. 2005), and second is the participation in tissue remodelling. Immunisation with purified YKL-39 induced arthritis in different strains of mice (Sakata et al. 2002). Histological examination revealed synovial proliferation and irregularity of the cartilage surface in BALB/c mice. After injection of purified YKL-39, not only anti-YKL-39 antibody was detected, but also the antibody against type II collagen, suggesting the spreading of autoimmune reactions (Sakata et al. 2002). This animal model suggested that the role of YKL-39 as an inducer of autoimmune processes related to arthritis. Antibodies to YKL-39 can be detected in human serum with ELISA and Western blotting, and were found in patients with rheumatoid arthritis (RA) and osteoarthritis (OA) (Sekine et al. 2001; Du et al. 2005; Tsuruha et al. 2002). Autoantibodies to YKL-39 were detected in 8–11.8% (Sekine et al. 2001; Tsuruha et al. 2002) of patients with RA, and in 11.1% (Tsuruha et al. 2002) of patients with OA, while only 1% of patients with RA had autoantibodies to YKL-40 (Sekine et al. 2001). The immune response to YKL-39 was independent of that to YKL-40 (Sekine et al. 2001). In patients with OA, the prevalence of autoantibodies to YKL-39 and other autoantigens on early stages of disease suggested that the autoimmune response occurs during the initial phase of cartilage degeneration (Du et al. 2005). Participation of YKL-39 in tissue remodelling was suggested on the basis of its high level in chondrocyte cultures and close homology to YKL-40 that was shown to induce cell proliferation and migration. However, the biological activity of YKL-39 remains to be identified.

### AMCase

Overproduction of enzymatically active AMCase was recently linked to asthma development (Elias et al. 2005; Zhu et al. 2004). Asthma is a chronic disease characterised by exaggerated Th2 airway inflammation (Ray and Cohn, 1999). Mouse model of ovalbumin-induced bronchial asthma has shown that the acid mammalian chitinase (AMCase) is involved in the pathophysiology of asthma and acts downstream of interleukin-13. Administration of anti-AMCase antibody leads to a decrease of Th2-inflammation, tissue eosinophilia and lymphocyte accumulation (Zhu et al. 2004). Similar effects were detected after the application of chitinase inhibitor allosamidin. Thus, the enzymatic activity of AMCase was suggested to be involved in asthma progression. Search for common genetic variants of human AMCase in pediatric asthma, revealed that both polymorphisms and haplotypes of AMCase are associated with bronchial asthma in children (Bierbaum et al. 2005). In murine models, AMCase was found to be a feature of anti-parasite responses. Thus, the finding that AMCase contributes both to the host anti-parasite response and asthmatic Th2-inflammation supports the concept that asthma is an abnormal anti-parasite response in the absence of the pathogen (Elias et al. 2005).

### SI-CLP

SI-CLP (stabilin-interacting chitinase-like proteins) is the most recent identified human Glyco_18 domain-containing protein (Kzhyshkowska et al. 2006b). It was found as an interacting partner and sorting ligand for the multifunctional receptor stabilin-1, which is specifically expressed on subpopulations of tissue macrophages and sinusoidal endothelial cells in liver, spleen, lymph node and bone marrow (Kzhyshkowska et al. 2006a, 2005, 2006c Martens et al. 2006). In parallel with stabilin-1, expression of SI-CLP mRNA was strongly upregulated in macrophages by the Th2 cytokine IL-4 and by dexamethasone. We developed rat monoclonal antibody 1C11 recognising N-terminal epitope in SI-CLP. This epitope is located upstream of conservative Glyco_18 domain and has no similarity with sequences of other human Glyco_18 containing proteins. Using the 1C11 antibody we demonstrated that IL-4 and dexamethasone in combination increase SI-CLP protein levels in macrophages, the extent of which varied between donors. Further, we found that macrophages treated with IL-4 secrete SI-CLP, while co-stimulation with dexamethasone blocked secretion and resulted in intracellular accumulation of SI-CLP. The 1C11 mAb recognised SI-CLP in the cellular fraction of bronchoalveolar lavage specimens obtained from patients with chronic inflammatory disorders of the respiratory tract and in PBLs from these patients as well as from healthy donors. Thus, 1C11 mAb can be applied for the examination of association of SI-CLP with human disorders.

SI-CLP is the only chitinase-like protein which is upregulated by glucocorticoids (Kzhyshkowska et al. 2006b). Interestingly, the highest expression level of SI-CLP was found by us in a patient with sarcoidosis undergoing corticoid therapy. We observed strong differences in stimulatory effect of dexamethasone on SI-CLP production in macrophages obtained from different healthy individuals. These facts indicate that SI-CLP is a promissing marker for the individual responsivity to glucocorticoids and prediction of side effects of corticoid treatment.

### YM1 and YM2

YM1 (ECF-L) and YM2 proteins do not exist in human. However, domain organisation and expression profile indicate that the function of murine YM1/2 can overlap with human AMCase and SI-CLP, and we will briefly describe the biological activity of YM1/2. Expression of YM1, similarly to AMCase and SI-CLP, is upregulated in Th2-driven immunological reactions. YM2 is very close homologue of YM1, and its expression depends on interleukin (IL)-4 and IL-13 signal transduction (Webb et al. 2001). YM1/2 are mainly associated with two pathological situations: parasite infections and lung disorders. During the experimental model of murine trypanosomosis, YM1 is expressed on the late stage of the infection characterised by the conversion of classical macrophage activation to the alternative one (Raes et al. 2002). Expression of YM1 is a generalised feature of nematode infection, and YM1 together with murine AMCase are highly upregulated in lung of mice infected with *Nocardia brasiliensis* (Nair et al. 2005). During the course of allergic peritonitis, macrophages secrete YM1 in IL-4 and STAT6-dependent manner (Welch et al. 2002). YM2 is strongly upregulated in the lung of BALB/c mice during OVA-induced allergic airways inflammation (animal model of asthma), where overproduction of YM2 was dependent on CD4+ T-cells and on signalling of IL-4 and IL-13 through the IL-4Rα subunit (Webb et al. 2001). Further, polymorphism in IL-4Rα in mice was shown to correlate with YM2 protein expression as well as with airways hypersensitivity and eosinophilia (Webb et al. 2001).

YM1, purified out of supernatant of splenocytes of C57BL6 mice was demonstrated to possess chemotactic activity towards eosinophils, T-lymphocytes and polymorphonuclear leukocytes in vitro, and to cause selective extravasation of eosinophils in vivo (Nio et al. 2004).

YM1 is crystallised in lung of motheaten mice, which are deficient in SHP-1 protein-tyrosine phosphatase activity resulting in a broad immunological dysregulations including hyperreactivity of alveolar macrophages (Guo et al. 2000). Progressive pulmonary injury in motheaten mice is characterised by consistent formation of intrapulmonary eosinophilic crystals. These crystals are morphologically similar, but biochemically not identical to the Charcot-Leyden crystals in human. Crystals of YM1 were also found within the aged lung at sites of chronic inflammation in the murine model of chronic granulomatosis disease (CGD) (Harbord et al. 2002). In human, CGD is a rare genetic disease caused by defects in NADPH oxidase and characterised by recurrent life-threatening bacterial and fungal infections. In murine model of CGD, YM1 was found in neutrophil granules, and its release during the acute inflammation caused the formation of extracellular crystals.

Both YM1 and Ym2 were found to be strongly upregulated in murine model of proliferative dermatitis, which is characterised by accumulation of eosinophils (Hogenesch et al. 2006). Overexpression of YM1/2 in the diseased skin was due to macrophages, dendritic cells, and mast cells, while no detectable amount of YM proteins was found in eosinophils and neutrophils (Hogenesch et al. 2006).

In conclusion, YM1/YM2 proteins participate in Th2-drived allergic processes, probably via induction of infiltration of inflammatory cells, including eosinophils. Whether human analogues of YM1/2proteins, i.e. AMCase and SI-CLP possess similar chemotactic activity remains to be investigated.

### Oviductin/MUC9

Exclusive secretion of oviductin/MUC9 by oviductal epithelium and its highest expression in time of ovulation gave basis for the suggestion of its regulatory role during fertilisation (Lok et al. 2002; Lapensee et al. 1997). It was hypothesised that the chitinase-like domain of oviductin targets it to specific oligosaccharide moieties in zona pellucida, where oviductin molecules might form protective shield around the oocyte (Malette et al. 1995). However, the studies performed in oviductin knock out mice failed to reveal abnormalities in fertilisation process (Araki et al. 2003). Specific function of oviductin in human remains to be established.

## Conclusions and perspectives

Enzymatically active chitinases chitotriosidase and AMCase; as well as chitinase-like proteins YKL-40, YKL-39 and murine YM1/2 have specific patterns of association with disorders. This pattern is defined by the cytokine-dependent expression of Glyco_18 domain-containing proteins by various cell types. Serum levels of chitotriosidase and YKL-40 reflect first of all the activation status of macrophages, and in particular lipid-laden macrophages. While chitotriosidase is a specific marker for lysosomal storage disorders, overexpression of YKL-40 was found in numerous of tumors, chronic and acute inflammations and during fibrosis progression. Neutrophils also contribute to the concentration of chitotriosidase and YKL-40 in the circulation. AMCase, SI-CLP and murine YM1/2 are expressed in Th2-environment, which was originally evolved for host-defence against parasites, while failure in control of Th2 reactions results in allergy and atopic asthma. Association of genetic markers of AMCase with bronchial asthma in children and animal aeroallergen asthma model indicate that AMCase is involved in asthma development; however, it is not clear whether epithelial cells and/or macrophage are crucial for the secretion of AMCase in lung. SI-CLP is produced by macrophages, T- and B-cells, as well as by tumor cells. However, secretion of SI-CLP was detected only in case of IL-4-stimulated macrophages in vitro, and further studies are needed to detect SI-CLP in the circulation. Novel rat monoclonal antibody against SI-CLP detects this protein with high specificity in human BAL samples as well as in peripheral blood leukocytes, and will serve as a tool for the examination of SI-CLP association with human disorders.

In conclusion, human Glyco_18 domain-containing proteins, can be used as major and supplementary markers for numerous inflammatory and malignant disorders. Comprehensive studies on large cohorts of patents are needed to establish the correlation of levels of Glyco_18 domains containing proteins with currently used clinical markers. Further studies are needed to elucidate the contribution of specific cell types in secretion of chitinases and chitinase-like proteins into the extracellular space and into the circulation. Generation of knockout animal models is needed to investigate the physiological role of Glyco_18 domain-proteins in the context of the whole organism. Understanding of physiological activity of chitinases and chitinase-like proteins as well as mechanisms of their cell-type specific secretion will allow the development of the cell-type specific therapeutic approaches.

## Figures and Tables

**Table 1:** Cell type specificity of expression of chitinases and chitinase-like proteins.

	Chitotriosidase (CHIT1)	AMCase (CHIA)	YKL-40 (CHI3L1)	YKL-39 (CHI3L2)	SI-CLP (CHID1)	*YM-1/2 (Chi3l3/l4)*
Macrophages (MΦ)	Mature, monocyte derived MΦ Gaucher cells; lung MΦ	Lung MΦ	MΦ at late stages of differentiation; MΦ stimulated with IFNγ; tumor-associated MΦ; MΦ and giant cells in pulmonary sarcoid granuloma; subsets of MΦ in the atherosclerotic plaque; *Microglia in AD*	*Microglia in AD*	MΦ stimulated with IL-4/dex; MΦ in BAL	*Microglia in AD murine model of chronic proliferative dermatitis; lung macrophages in IL-13- transgenic mice*
Neutrophils	PMN of healthy donors		Non-activated and activated neutrophils			*Neutrophils murine model of CGD*
Mast cells						*Murine model of chronic proliferative dermatitis*
T-cells					Jurkat cells, CD3+ cells from peripheral blood	
B-cells					Raji cells	*B-cells in draining lymph node of mice implanted with Brugia malayi*
Epithelial cells		*Non- mucus- producing epithelial cells of distal airway*				*Mucus- producing epithelial cells of distal airway*
Synovial cells			Fibroblast-like synovial cells	Fibroblast cells like synovial cells		
Chondrocytes			RA and OA chondrocytes	Articular cartilage chondrocytes		
Smooth- muscle cells			Vascular smooth-muscle cells (VSMC)			
Malignant tumors and tumor cell lines			Glioblastoma (U87); osteosarcoma (MG-63); ovarian cancer (SW626); prostate cancer (DV-145); malignant melanoma (SK-MEL-28); cancer cells in biopsies of glioblastoma, breast and prostate cancer		HEK293, MCF7, HeLa, A-549	

The expression profile is described predominantly for human proteins. Expression in mouse cells is indicated in italic.

**Table 2:** Chitinases and chitinase-like proteins: biological activity.

	Enzymatic activity	Proliferative effect	Effect on cell adhesion and migration	Immunomodulatory effect
Chitotriosidase	Hydrolytic and *trans-* glycosylation activity			
AMCase	Hydrolytic activity			Regulation of IL-13 downstream effect in asthma
YKL-40	No	Growth- promoting effect on human synovial cells, skin and foetal lung fibroblasts	Migration, spreading and attachment of VSMC	In healthy donors: induction of IL-10 production in monocytes and T-reg; in RA patient: induction of Th1 immune response; suppression the TNFα and IL-1-induced secretion of MMPs and IL-8 in human skin fibroblasts and articular chondrocytes
YKL-39	No			Induction of autoimmune processes (Sakata et al. 2002; Tsuruha et al. 2002)
YM1/YM2	No		Chemotaxis for eosinophils, T-cells, polymorphonuclear leukocytes	

**Table 3:** Chitinases and chitinase-like proteins: association with disorders.

	Infectious diseases	Lysosomal storage diseases	Chronic inflammation	Allergy	Cancer	Liver disorders	Neurodegenerative diseases
Chitotriosi Dase	Fungal and, bacterial; malaria	Gaucher disease; Niemann- Pick disease; Fabry disease	Atherosclerosis; sarcoidosis			Non- alcoholic fatty liver disease – steatohepatitis (NASH)	Alzheimer’s disease; ischemic cerebro vascular dementia
AMCase				Asthma			
YKL-40	Pneumonia; purulent meningitis; *Escherichia coli* endoto xaemia		Atheroscleosis; rheumatoid arthritis; sarcoidosis; inflammatory bowel disease		Diagnostic and prognostic marker for number of solid tumors	HCV- induced liver fibrosis and cirrhosis, fibrosis in alcoholics	
YKL-39			Osteoarthritis; rheumatoid arthritis				
SI-CLP				Th2-induced pathologies suggested			
YM1/YM2	Parasite Infections in rodents						
